# Evidential Proximity, Independence, and the evaluation of carcinogenicity

**DOI:** 10.1111/jep.13226

**Published:** 2019-07-09

**Authors:** Jon Williamson

**Affiliations:** ^1^ Department of Philosophy University of Kent Canterbury UK

**Keywords:** causality, epistemology, evaluation, philosophy of medicine, public health

## Abstract

This paper analyses the methods of the International Agency for Research on Cancer (IARC) for evaluating the carcinogenicity of various agents. I identify two fundamental evidential principles that underpin these methods, which I call Evidential Proximity and Independence. I then show, by considering the 2018 evaluation of the carcinogenicity of styrene and styrene‐7,8‐oxide, that these principles have been implemented in a way that can lead to inconsistency. I suggest a way to resolve this problem: admit a general exception to Independence and treat the implementation of Evidential Proximity more flexibly where this exception applies. I show that this suggestion is compatible with the general principles laid down in the 2019 version of IARC's methods guide, its *Preamble to the Monographs*.

## 1 INTRODUCTION

The International Agency for Research on Cancer (IARC) is the World Health Organization body responsible for evaluating the carcinogenicity of environmental and occupational exposures. These include a wide range of individual chemicals, as well as mixtures such as welding fumes and behavioural factors such as shift work. Since the start of the programme in 1970, over a thousand such agents have been evaluated, many of them more than once due to new evidence becoming available.

IARC is interesting from the point of view of the methodology of evidence evaluation because it is one of the few organizations that systematically evaluates a very broad evidence base—including epidemiological studies on humans, (nonhuman) animal studies, and mechanistic studies—and it has developed an explicit recipe for integrating the results of the evaluation of each sort of study. Clarke et al^1^ argue that there is a tendency elsewhere in biomedicine, propagated by the evidence‐based medicine movement, to focus in the final assessment of causation almost exclusively on statistical studies on humans and to disregard or downplay other sorts of study and that this tendency is problematic. IARC is notable for not succumbing to this tendency.

In Section 2, I analyse IARC's evaluation procedures and argue that they conform to the following two general evidential principles:

*Evidential Proximity*: Studies on populations closer to the population of interest carry more weight than studies on more remote populations, ceteris paribus.
*Independence*: Each agent is assessed solely on the basis of evidence specific to that agent.

While these epistemological principles are well motivated, I shall show in Section 3 with reference to the 2018 evaluations of styrene and styrene‐7,8‐oxide that the way they are currently implemented in IARC procedure can in principle lead to inconsistent assessments of carcinogenicity. In Section 4, I propose improvements to the procedure that would avoid this source of error. Finally, in Section 5, I show that these suggestions are compatible with IARC's new (2019) articulation of its general principles for evaluating carcinogenicity.^2^ Thus, the proposal is for changes to the implementation of the general principles rather than to the general principles themselves. *
I should emphasize that the focus of this paper is on methodology; I do not criticize any particular past assessment of carcinogenicity.


## 2 EVIDENTIAL PRINCIPLES UNDERLYING IARC's METHODS

IARC's goal is to determine *whether* the agent in question is a cause of cancer and the sites within the body where cancers occur, not the *extent* to which the agent causes cancer. This is called determining “cancer hazard” rather than “cancer risk.” The rationale for this focus is that exposure to the agent in question varies geographically and over time, and so cancer risk varies. It is left up to individual governments to determine whether to restrict an agent whose carcinogenicity is established or likely. This decision typically depends not only on cancer hazard but also on the assessment of local risk, the impact on local businesses and, alas, the influence of industries that sell or propagate the agent in question.

IARC is interesting from the point of view of the methodology of evidence evaluation because it is one of the few organizations that has a detailed procedure for systematically evaluating mechanistic evidence.

Clarke et al [1, Section^1^] argue that in recent decades, there has been a widespread tendency in biomedicine—promoted by the evidence‐based medicine movement—to focus on evaluating clinical and epidemiological studies and to disregard or downplay other sources of evidence, such as expert opinion, basic science, and mechanistic studies. A clinical or epidemiological study for the claim that *A* is a cause of *B* in a target population of interest measures the incidence of *A* and *B* in populations close to the target population, together with possible confounding factors that might explain away an observed correlation between *A* and *B*. When evaluating medicines, randomized studies are common. In the assessment of environmental and occupational exposures, however, it is normally only feasible to conduct *observational* studies on populations close to the target population, for ethical reasons; experimental studies remain ethically permissible in populations involving animal models, which are somewhat remote from the target human population. Clinical and epidemiological studies help to determine *whether A* causes *B*, not *how A* causes *B*. Mechanistic studies, in contrast, seek to elucidate features of the mechanism by which *A* might cause *B*.

**Figure 1:**
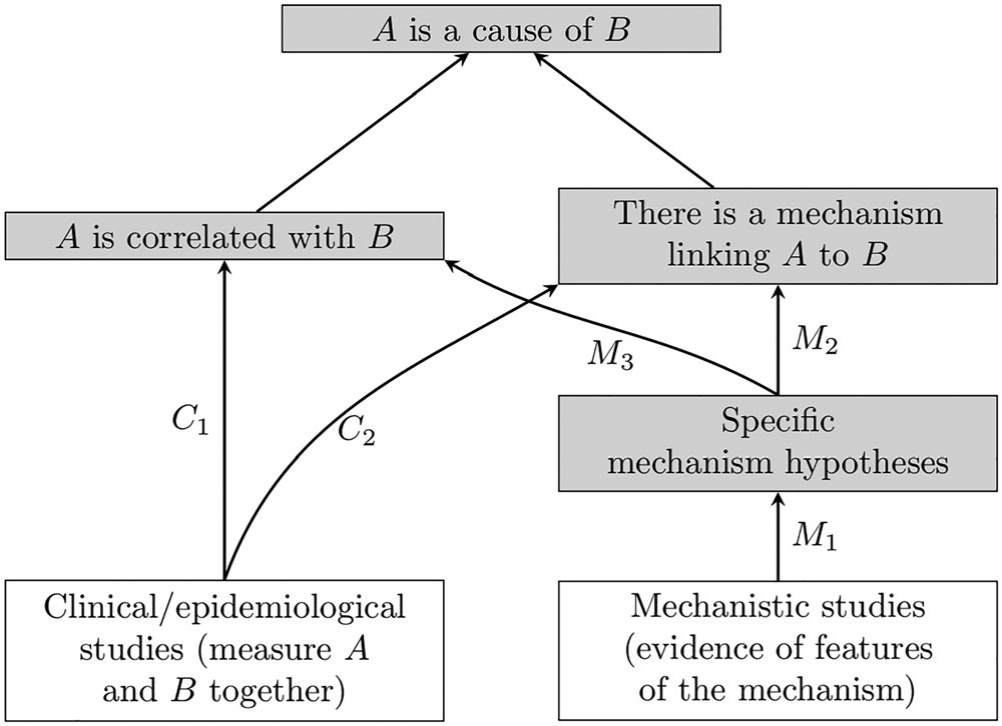
Evidential relationships for evaluating a causal claim^3^

The tendency to disregard or downplay mechanistic studies may be attributable to the fallacious inference that mechanistic studies help to determine *how*, not *whether*, *A* causes *B*. In fact, mechanistic studies can be crucial to the evaluation of whether *A* causes *B*. Figure 1, from Williamson,^3^ provides a conceptualization of the problem that explains why this is the case. As is well recognized, correlation is insufficient for causation. What distinguishes a causal connection from other explanations of a correlation, such as confounding, bias or chance, is the existence of a mechanism linking *A* to *B*, which explains instances of *B* in terms of *A* and which can account for the extent of the observed correlation. Accordingly, to establish that *A* is a cause of *B*, one needs to establish not only that *A* is correlated with *B* but also that there exists some mechanism of action.^4^ Clinical or epidemiological studies are the primary means of confirming a correlation in biomedicine; this channel of confirmation is labelled *C*_1_ in Figure 1. Clinical and epidemiological studies can also, in the right circumstances, establish the existence of a mechanism (*C*_2_): If sufficiently many studies consistently observe a large enough correlation and their study designs are of sufficiently high quality, then one may be able to rule out noncausal explanations of the correlation, such as confounding, bias, and chance, and infer that there must be some mechanism that gives rise to the correlation, even if the details of the mechanism remain unknown. However, another way to confirm the existence of a mechanism of action involves finding key features of that mechanism (*M*_2_), and these features are in turn confirmed by mechanistic studies (*M*_1_). In addition, features of the mechanism can also confirm the existence of a correlation (*M*_3_). However, clinical and epidemiological studies, where they exist, are usually more informative in this regard.

Returning to the evaluation of carcinogenicity, epidemiological studies often fail on their own to provide conclusive evidence of carcinogenicity along the *C* channels. This leaves room for mechanistic studies to influence the overall evaluation along the *M* channels. In several cases, IARC's preliminary evaluation of carcinogenicity, formed on the basis of human and animal studies, has been upgraded by confirmatory evidence from mechanistic studies—this happened in the case of benzo[a]pyrene, for example.^5^ In other cases, the preliminary evaluation of carcinogenicity has been downgraded by disconfirming evidence from mechanistic studies—this happened in the case of *d*‐limonene, for example.^6^

**Figure 2:**
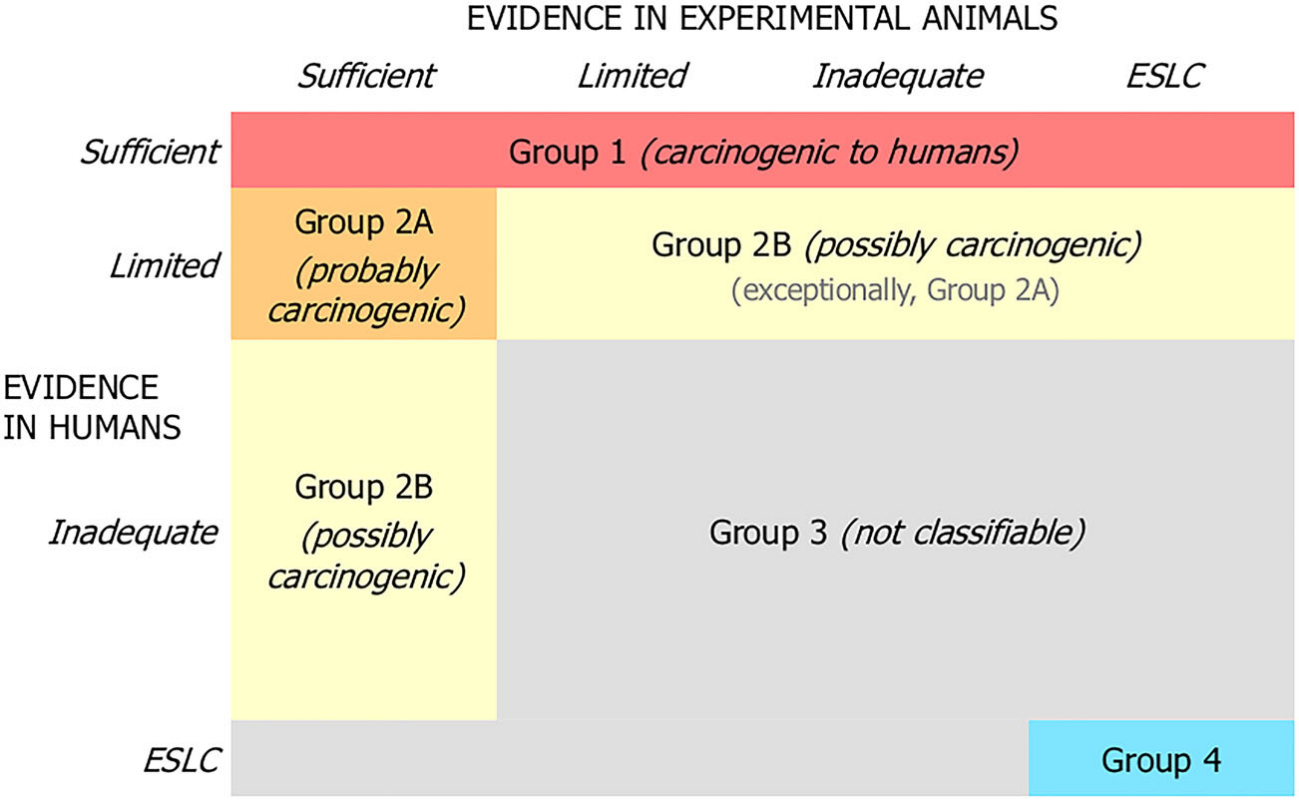
The International Agency for Research on Cancer's (IARC's) use of human and animal studies, as of 2018 (http://monographs.iarc.fr/ENG/Publications/Evaluations.pdf)

**Figure 3:**
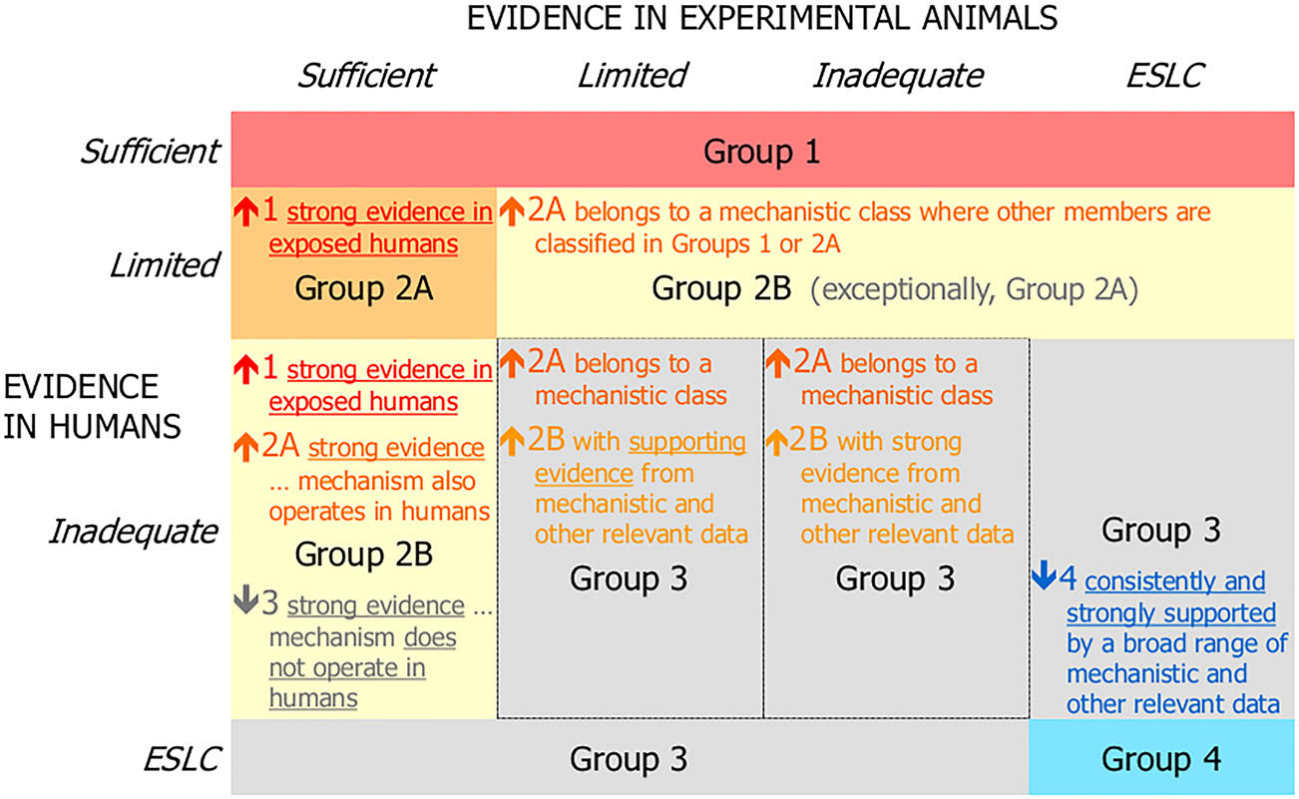
The International Agency for Research on Cancer's (IARC's) use of mechanistic studies and other considerations to upgrade and downgrade evaluations, as of 2018 (http://monographs.iarc.fr/ENG/Publications/Evaluations.pdf)

IARC's procedure is constantly evolving. The “Preamble” to IARC's monographs on carcinogenicity encodes the general principles of its procedure, but long periods pass between revisions of this document, and the way in which the general principles are implemented can change more rapidly. IARC^7^ recorded the general principles as of 2006, and this version of the Preamble was in use until 2019, when revisions were captured in a new version of the Preamble.^2^ This paper is particularly concerned with some agents evaluated in 2018, and the methods in use at that evaluation can be summarized as follows. First, assessments of human and animal studies are used to form a preliminary evaluation of carcinogenicity. Figure 2 explains this evaluation. Carcinogenicity is evaluated on the following scale: 1 (the agent is carcinogenic to humans), 2A (it is probably carcinogenic to humans), 2B (it is possibly carcinogenic to humans), 3 (it is not classifiable as to its carcinogenicity to humans), and 4 (it is probably not carcinogenic to humans). Human and animal studies are assessed on the following scale: sufficient (causation is established), limited (minor limitations to the evidence), inadequate (major limitations to the evidence), and ESLC (evidence suggesting lack of carcinogenicity). For instance, inadequate studies in humans and sufficient studies in animals would yield a preliminary rating of *possibly carcinogenic*, 2B. Next, mechanistic studies are taken into account, and this can lead to the preliminary rating being upgraded or downgraded, as explained in Figure 3. The mechanistic studies are rated as strong, moderate, or weak according to how strongly they confirm 10 standard specific mechanistic hypotheses, called the “ten key characteristics of carcinogens”,^8^ eg, the hypothesis that the agent is genotoxic or the hypothesis that it is electrophilic. Then, an assessment is made as to whether a hypothesized mechanism is likely to be operative in humans. If it is, an upgrade of the preliminary evaluation may be possible—eg, from 2B to 2A. On the other hand, if there is evidence that the mechanism does not operate in humans, then a downgrade is possible, as happened with *d*‐limonene.

Figures 2 and 3 support the following observations. First, evidence in humans is treated as more decisive than evidence in animals. Evidence in humans graded as “sufficient” is enough to establish carcinogenicity, for instance. Second, mechanistic studies have a relatively minor influence. For example “sufficient” evidence in animals is normally enough for a 2B rating, but mechanistic evidence on its own, without any evidence from humans or animals, would not yield anything more than 3 (unclassifiable).

One thing that is not apparent from these tables, but is set out more clearly in IARC's Preamble and is clear in discussions of the working group that considers mechanistic studies, is that the mechanistic studies themselves have varying influence. IARC^7, p21^ notes that “the strongest indications that a particular mechanism operates in humans derive from data on human or biological specimens obtained from exposed humans.” In fact, in vivo studies in exposed humans carry most weight, ceteris paribus, followed by ex vivo studies (where specimens are extracted from exposed humans for experimentation and analysis), then in vitro studies on human cells, then in vivo studies in mammals, with higher weight given to studies involving studies mechanistically closer to humans, followed by studies on nonmammals and model organisms, followed by studies involving toxicokinetic and pharmacokinetic models (which are mathematical models).

Arguably, then, IARC's procedures can be said to conform to the following evidential principle. When assessing whether a causal claim holds of a population of interest,

*Evidential Proximity*. Studies on populations closer to the population of interest carry more weight than studies on more remote populations, ceteris paribus.

IARC is interested in causes of cancer in humans, so the population of interest is the population of humans. Here, I take it that one population is close to another if it is mechanistically close: if the mechanisms that are relevant to the causal claim are similar. Now, two mechanisms can be said to be similar when key features of the mechanisms (ie, key entities, activities, and structural features) are similar (ie, are similar enough for one to be able to infer that the mechanisms as a whole have a similar function). The ceteris paribus condition ensures that high‐quality studies on a more remote population can, in the right circumstances, have more weight than poor quality studies on a closer population. One can justify Evidential Proximity by observing that mechanistic similarity is required to successfully extrapolate a causal claim from one population to another.^9^, ^10^ Ceteris paribus, results of studies are more likely to extrapolate to humans the closer the mechanisms of action in the study are to those operational in humans.

A second principle is apparent from IARC's protocol. †
As with Evidential Proximity, this principle is not exclusive to IARC. Arguably, the Independence principle is embedded in all evaluations made on the basis of systematic review. A review protocol excludes from consideration studies and evaluations that are not specific to the causal relationship under investigation. With one kind of exception, discussed below, each agent is assessed on its own merits:

*Independence*. Each agent is assessed solely on the basis of evidence specific to that agent.

The evidence specific to an agent includes human and animal studies that investigate exposure to that agent, as well as mechanistic studies that shed light on features of mechanisms involving that agent. The Independence principle ensures that the evidence base relevant to one agent is evaluated independently of the evaluations of other agents. A particular study may investigate two agents and may feature in the evidence base of each agent. However, the two evidence bases are then evaluated independently: The evidence base for one agent *screens off* the evaluation of the other. Independence accords well with Evidential Proximity. Studies of the carcinogenicity of other agents are somewhat remote from the question of whether the agent under investigation is carcinogenic, because different mechanisms are likely to operate, so the evaluations of the other agents should not influence that of the agent in question, given its evidence base. There is one exception to the Independence principle that is embedded in IARC procedure. This the case when the agent being evaluated is in a class of agents that have the same mechanism of carcinogenicity and some of which have been previously classified as carcinogenic or probably carcinogenic. In that case, we see in Figure 3 that these previous evaluations can make a difference: The rating of the agent can be upgraded from 2B to 2A.

## 3 A PROBLEM CASE: STYRENE AND STYRENE‐7,8‐OXIDE

IARC has evaluated the carcinogenicity of styrene and styrene‐7,8‐oxide three times each, most recently in March 2018.^11^ The 2018 evaluation of these agents is interesting because it suggests a problematic case for IARC procedure.

Styrene is a colourless volatile liquid, produced mainly in East Asia and used in the production of polystyrene and other compounds that feature in a diverse range of goods, including LEGO blocks, boats, and wind‐turbine blades. Styrene is inhaled in tobacco smoke, air pollution, from food packaging materials, and in the manufacturing process. In the recent evaluation, human evidence was judged *limited*, but the animal evidence was deemed *sufficient*. This combination leads to a preliminary overall classification of 2A (see Figure 2).

Styrene‐7,8‐oxide is a clear liquid and widely produced. It is used in epoxy resins and in compounds that are key to the production of many chemicals. Little is known about exposure other than that it is inhaled in the reinforced plastics industry. In the recent evaluation, human evidence was judged *inadequate*, but animal evidence was *sufficient*. This leads to a preliminary overall classification of 2B (Figure 2).

There were also a large number of mechanistic studies relevant to the 10 key characteristics of carcinogens that are routinely evaluated by IARC. As we have seen, these can moderate the overall classification by supporting the claim that there is a mechanism of action in humans. In addition, some mechanistic studies supported a further mechanistic assertion. It turns out that styrene is readily oxidized to form styrene‐7,8‐oxide and that, by far the most plausible route of carcinogenicity proceeds via this oxide (indeed, the mechanisms working group ruled out the main alternative hypothesis: the hypothesis that carcinogenicity proceeds via 4‐vinylphenol). The assertion, then, is this:

- Styrene only causes cancer, if it does at all, via the intermediary styrene‐7,8‐oxide.

Here is the problem. Human and animal evidence led to a preliminary rating of 2A for styrene and 2B for styrene‐7,8‐oxide. This classes styrene as probably carcinogenic and styrene‐7,8‐oxide as possibly carcinogenic but *not* probably carcinogenic. However, the probability of styrene‐7,8‐oxide being carcinogenic must be at least that of styrene being carcinogenic, given (A). We have a contradiction. ‡
The concern here is that inconsistency *can* arise, not that it *did* arise in this particular evaluation. While the preliminary overall classification would have led to inconsistency, in the final evaluation of styrene and styrene‐7.8‐oxide mechanistic studies were deemed in each case to provide strong evidence for the existence of a mechanism of carcinogenicity in humans. This made no difference to the preliminary evaluation of styrene, which was 2A, but meant that styrene‐7,8‐oxide was upgraded from 2B to 2A (cf. Fig. 3). Fortuitously, inconsistency was avoided in the final evaluation.


## 4 PROPOSED RESOLUTION

In order to avoid the possibility of contradiction in this example, one would want to reason as follows: There is inadequate evidence specific to styrene‐7,8‐oxide, but (A) holds and there is stronger evidence specific to styrene of its carcinogenicity, hence the evaluation of styrene‐7,8‐oxide should be influenced by that of styrene (it should be raised from 2B to 2A). However, Independence and Evidential Proximity, as currently implemented, conspire to thwart this inference.

It is primarily Independence that ensures that one cannot upgrade the evaluation of styrene‐7,8‐oxide in the light of (A) and strong evidence of the carcinogenicity of styrene. This is because the evaluation of styrene is screened off from that of styrene‐7,8‐oxide by the evidence base specific to styrene‐7,8‐oxide (similarly, one cannot use the evaluation of styrene‐7,8‐oxide to downgrade the evaluation of styrene in order to ensure consistency). Note that the one exception to Independence does not apply here: Styrene and styrene‐7,8‐oxide are not in a class of structurally similar agents, some of which have previously been classified as carcinogens—rather, one is an intermediary along the pathway from the other to carcinogenicity.

It is not even possible for (A) on its own to influence the evaluation of styrene‐7,8‐oxide, given Independence as currently implemented. According to current IARC protocol, the influence of mechanistic studies is mediated by the 10 key characteristics of carcinogens (unless the mechanistic class hypothesis applies). However, (A) sheds no light on any pathway from styrene‐7,8‐oxide to cancer, so (A) says nothing about the 10 key characteristics of carcinogens, insofar as they apply to styrene‐7,8‐oxide. Thus, not only is the evaluation of styrene screened off from that of styrene‐7,8‐oxide, assertion (A) itself is deemed irrelevant to the evaluation of styrene‐7,8‐oxide, on current IARC procedure.

Furthermore, we saw above that Evidential Proximity yields a strict hierarchy of mechanistic evidence. Indeed, IARC implements Evidential Proximity in such a way that for mechanistic studies to be evaluated as providing *strong* evidence that there is a mechanism operational in humans, there need to be convincing mechanistic studies *on humans*. However, low‐level mechanistic assertions like (A), dealing with the way in which compounds are metabolized, need not be established on the basis of (often observational) studies on humans. Often, experimental studies in the lab are the primary grounds for such claims—studies that would typically be classed by IARC as providing weak mechanistic evidence. Thus, IARC's implementation of Evidential Proximity provides no natural place for evidence in favour of (A) to influence the evaluation of carcinogenicity.

In order to eradicate the potential for inconsistency, one thus needs to alter the implementation of IARC's procedures. One might suggest dropping Evidential Proximity or Independence or both. However, these are both plausible principles, and there is no need to abandon either in a wholesale way. A change to the implementation of each of these principles would remedy the situation.

First, the implementation of Independence must change. The evidence base specific to styrene‐7,8‐oxide included human and animal studies—which on their own were rather inconclusive—as well as mechanistic studies that establish assertion (A). Now, (A) itself provides grounds for looking beyond the evidence base specific to styrene‐7,8‐oxide: It implies that the evaluation of styrene could be relevant to that of styrene‐7,8‐oxide. Therefore, the evidence base specific to styrene‐7,8‐oxide motivates a new exception to Independence.

One might try to alter Independence, then, by adding another explicit exception. However, it is far from clear as to how to formulate this second exception in a way that is both general enough to be applicable in further cases yet specific enough as to be plausible. One might try “if the agent can only cause cancer via a pathway upon which lies a second agent whose carcinogenicity has been considered, the classification of the first agent should not exceed that of the second agent.” But the second agent may have been evaluated at a time when less evidence was available, in which case the inconsistency may reflect this change of evidence and, if so, would be less troubling than the inconsistency identified above. In this case, upgrading the rating of the second agent would not be an option unless the second agent were under consideration for re‐evaluation. Any explicit formulation of this second exception to Independence will clearly be rather complicated. Moreover, it is likely that further exceptions will be needed to deal with similar situations in which where there are multiple causal pathways to carcinogenicity. The worry is that this proposal would increase the number of ad hoc exceptions to a core principle, would be complex, and the list of exceptions may yet remain incomplete.

The exception to Independence can, however, be pitched at a general level:

*Independence*: Each agent should be assessed solely on the basis of evidence specific to that agent, *unless there is mechanistic evidence in that evidence base that warrants taking other evaluations into account.*

The advantage of this formulation is that it encompasses the mechanistic class exception (where the evidence base includes mechanistic evidence that the agent is in a class of agents established to cause cancer in the same way) as well as the mechanistic pathway exception—eg, assertion (A)—and more complex variants of this exception. By reformulating Independence in this way, we avoid the need for a list of concrete exceptions that would appear both ad hoc and potentially incomplete.

In addition, the implementation of Evidential Proximity must also change. In IARC's methodology as outlined above, mechanistic studies in humans on the agent in question are required for the mechanistic evidence to count as “strong” and to warrant a substantial upgrade in the preliminary evaluation of an agent. This restriction is appropriate in relation to evidence for the key characteristics of carcinogens, but it is not necessarily appropriate when considering the exceptions to Independence. That an agent is a member of a mechanistic class may be established by bench work in the laboratory—studies on humans or animals may not be required. Similarly, a pathway hypothesis such as (A) may be established by studies other than those carried out on humans. In sum, nonhuman mechanistic studies may be sufficient to establish specific mechanistic hypotheses other than the 10 key characteristics of carcinogens. The formulation of Evidential Proximity of Section 2 remains intact, but its implementation needs to depend on the specific mechanistic hypothesis under consideration.

The proposal is, then, that Independence and Evidential Proximity should be implemented more flexibly. Independence requires a general exception and, when considering evidence relevant to this exception, Evidential Proximity should not invariably be taken to require evidence obtained on humans. IARC working groups responsible for the evaluation of mechanistic studies would be expected to focus on the 10 key characteristics of carcinogens, and on evidence in humans, unless there is compelling evidence for some other specific mechanistic hypothesis, such as the mechanistic class hypothesis or a mechanistic pathway hypothesis analogous to (A). There will be others, no doubt. There is no need to formulate every such hypothesis in advance as an explicit exception. Mechanistic evidence influences assessments of causation in heterogeneous ways,^12^ and it suffices that the mechanistic working group be receptive to new possibilities.

## 5 IARC's REVISED PREAMBLE

Thus far, I have suggested that the problem posed by styrene and styrene‐7,8‐oxide can be mitigated by increasing the range of specific mechanism hypotheses that can influence the overall classification of an agent. This change can be thought of as replacing an ad hoc exception to the Independence principle by a general exception and by implementing Evidential Proximity in a more nuanced way. It turns out that IARC's new Preamble^2^ leaves some room for this additional flexibility, as we shall now see.

Perhaps the main innovation in the 2019 Preamble is with regard to the treatment of mechanistic studies. Mechanistic studies are now treated on a par with human studies and animal studies, rather than having a subsidiary role whereby human and animal studies determine a preliminary classification as per Figure 2, and mechanistic studies can be used to make small adjustments to the final classification (Figure 3). This change reflects increasing recognition that, in general, mechanistic evidence should be treated on a par with clinical and epidemiological studies when assessing causality (Figure 1).

The Preamble now explicitly acknowledges the centrality of the 10 key characteristics of carcinogens to the evaluation of mechanistic studies.^2, section B.4.b^ (The Preamble emphasizes that these 10 characteristics are not detailed descriptions of mechanistic pathways from the agent to cancer but are key features of such pathways that are indicative of carcinogenicity). Importantly, the 2019 Preamble also provides room for “evidence that falls outside of the recognized key characteristics of carcinogens, reflecting emerging knowledge or important novel scientific developments on carcinogen mechanisms.”^2, p32^ While this provision is chiefly intended to allow for revisions to the list of key characteristics of carcinogens in the period before the next update of the Preamble, it can also be construed as providing space for specific mechanism hypotheses other than the key characteristics, such as assertion (A). Thus, the general exception to Independence, outlined above, is compatible with the approach to mechanistic studies set out in the 2019 Preamble. Likewise, although the new Preamble does not explicitly mention specific mechanism hypotheses other than the 10 characteristics of carcinogens and the mechanistic class hypothesis, it only requires studies on humans when classifying mechanistic evidence for the key characteristics of carcinogens as strong. This leaves open the possibility that a mechanistic pathway hypothesis such as (A) can be treated analogously to the mechanistic class hypothesis. Thus, the recommendations put forward in this paper are compatible with IARC's general principles as currently formulated.

**Figure 4:**
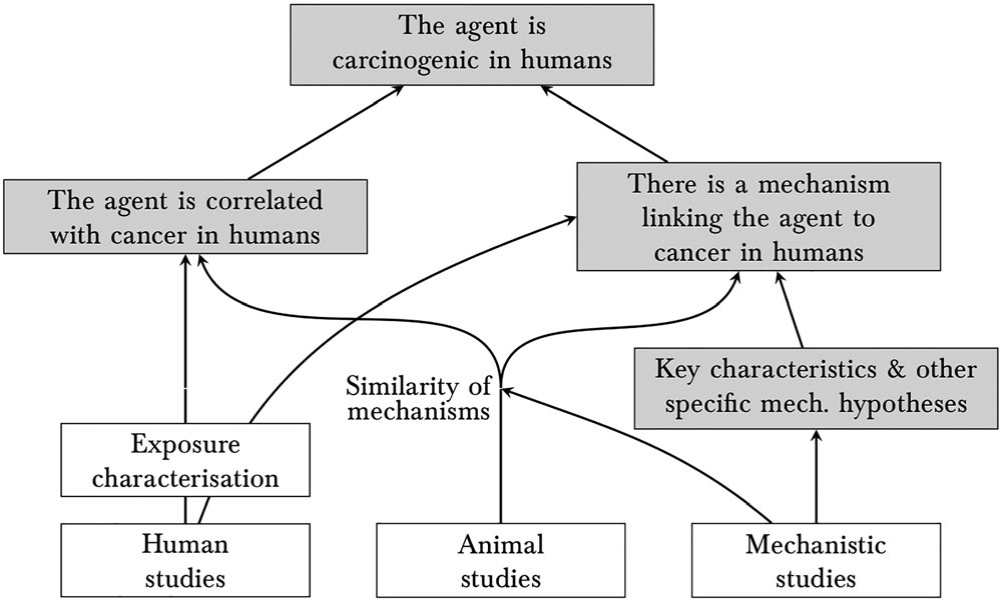
Evidential relationships for the International Agency for Research on Cancer's (IARC's) evaluations

In the light of Figure 1, the evidential relationships at the core of IARC's 2019 methodology can be conceptualized by means of Figure 4. The team responsible for an evaluation encompasses four working groups, looking at exposure characterization, human studies, animal studies, and mechanistic studies, respectively. Human studies, in the light of what is known about exposure, can directly test the claim that the agent under investigation is correlated with cancer in humans. If it is, and if there are sufficiently many high quality studies, and if these studies are sufficiently concordant, they can also confirm the general mechanistic claim that there exists some mechanism of carcinogenicity in humans that is responsible for this correlation, thereby confirming the overall causal claim. Animal studies can also confirm this correlation claim and general mechanistic claim as long as it is established that the mechanisms of carcinogenicity in those animals are sufficiently similar to those in humans. In addition, mechanistic studies test the general mechanistic claim via the key characteristics of carcinogens. Crucially, other specific mechanism hypotheses, such as (A), may also be relevant to the truth of the general mechanistic claim, and the proposal is that they should be taken into account where applicable. Such a hypothesis can motivate moving beyond the evidence base of the agent under investigation: Thanks to (A), for example, the evaluation of styrene is relevant to the assessment of styrene‐7,8‐oxide and should influence its overall evaluation. This requires no change to the overall picture—just a recognition that “other specific mech. hypotheses” can include hypotheses such as (A) in addition to the mechanistic class hypothesis.

## 6 CONCLUSION

In this paper, I have argued that IARC's procedures conform to two general principles, Evidential Proximity and Independence. Both of these principles are plausible, from an epistemological point of view. Recent work on extrapolation provides support for Evidential Proximity, and Independence captures the evidentialist precept that studies relevant to a causal claim should screen off the evaluation of that claim from other causal judgements.

We saw that the implementation of these general principles needs some care. The case of styrene and styrene‐7,8‐oxide poses a problem for the current implementation of IARC's rules for assessing carcinogenicity. This is a problem readily solved, however. Incoherence can be avoided by formulating a general exception to Independence and implementing Evidential Proximity more flexibly where this exception applies.

The points made here readily generalize to other situations in which causal claims are systematically evaluated. Evidential Proximity and Independence remain credible when assessing other forms of exposure and when assessing the effectiveness of interventions. In any such situation, it is hard to limit in advance the range of specific mechanism hypotheses that might be relevant. Rather, the pertinent specific mechanism hypotheses can be expected to vary from causal claim to causal claim.^12, section^ ^5^ ^.1^

## CONFLICT OF INTEREST

The authors declare no conflict of interest.

## References

[1] Clarke B , Gillies D , Illari P , Russo F , Williamson J . Mechanisms and the evidence hierarchy. Topoi. 2014;33(2):339‐360.

[2] IARC . IARC monographs on the evaluation of carcinogenic hazards to humans: Preamble. Lyon 2019 https://monographs.iarc.fr/wp-content/uploads/2019/01/Preamble-2019.pdf.

[3] Williamson J . Establishing the teratogenicity of Zika and evaluating causal criteria. Synthese. 2018;1‐14. 10.1007/s11229-018-1866-9 30872868

[4] Russo F , Williamson J . Interpreting causality in the health sciences. International Studies in the Philosophy of Science. 2007;21(2):157‐170.

[5] IARC . Chemical agents and related occupations; 100F of IARC Mongraphs Series Lyon: International Agency for Research on Cancer 2012 http://monographs.iarc.fr/ENG/Monographs/vol100F/mono100F.pdf.

[6] IARC . Some chemicals that cause tumours of the kidney or urinary bladder in rodents and some other substances; 73 of IARC Monographs Series. Lyon: International Agency for Research on Cancer 1999 http://monographs.iarc.fr/ENG/Monographs/vol73/mono73.pdf.

[7] IARC . IARC monographs on the evaluation of carcinogenic risks to humans: Preamble. Lyon 2006 https://monographs.iarc.fr/wp-content/uploads/2019/01/Preamble_updated2015.pdf.

[8] Smith MT , Guyton KZ , Gibbons CF , et al. Key characteristics of carcinogens as a basis for organizing data on mechanisms of carcinogenesis. Environmental Health Perspectives. 2016;124(6):713‐721. 26600562 10.1289/ehp.1509912 PMC4892922

[9] Steel D . Across the Boundaries: Extrapolation in Biology and Social Science. Oxford: Oxford University Press; 2008.

[10] Parkkinen VP , Williamson J . Extrapolating from model organisms in pharmacology In: La Caze A, Osimani B, eds. Uncertainty in Pharmacology: Epistemology, Methods, and Decisions. Dordrecht: Springer; 2017.

[11] IARC . Carcinogenicity of quinoline, styrene, and styrene‐7,8‐oxide. The Lancet Oncology. 2018;19:728‐729. 10.1016/S1470-2045(18)30316-4 29680246

[12] Parkkinen VP , Wallmann C , Wilde M , et al. Evaluating evidence of mechanisms in medicine: principles and procedures. Cham, Switzerland: Springer; 2018. 31314227

